# Gastrosplenic Fistula without Malignancy Management in a 16-Year-Old Boy

**DOI:** 10.1055/s-0039-1678568

**Published:** 2019-12-13

**Authors:** Aila Malik, Chinwendu Onwubiko, Mike Chen, Andrei Radulescu, David Galloway, Colin Martin

**Affiliations:** 1 Medical College, CMH Lahore Medical College and Institute of Dentistry, Lahore, Pakistan; 2 Department of Surgery, University of Alabama at Birmingham, Birmingham, Alabama, United States; 3 Department of Surgery, Loma Linda University Adventist Health Sciences Center, Loma Linda, California, United States; 4 Department of Pediatrics, University of Alabama at Birmingham, Birmingham, Alabama, United States

**Keywords:** gastrosplenic fistula, subtotal gastrectomy, splenic infarct, pancreatitis

## Abstract

Gastrosplenic fistula is a very rare entity, most commonly occurring as a distinctive complication of splenic or gastric malignancies, most notably diffuse large B cell lymphoma (DLBCL). Benign gastric ulcer, splenic abscess, and Crohn's disease have also been reported as possible causes. We report a nonmalignant case of 16-year–old male with a gastrosplenic fistula of unclear etiology. The fistulous tract was confirmed by an upper endoscopy and an upper gastrointestinal series. Subsequently, it was surgically managed with a subtotal gastrectomy with “Roux-en-Y” reconstruction and a feeding jejunostomy.

## Introduction

A gastrosplenic fistula is a very rare pathological condition. It was first described in two patients with splenic lymphoma by de Scoville et al in 1962. 1 “Aerosplenomegaly” is the radiological term used to describe an enlarged spleen, which appeared to be filled with air, which would suggest an enterosplenic fistula. A gastrosplenic fistula can form as a complication of a variety of conditions, although splenic lymphoma has most frequently been reported in previous literature. 2 We discuss a nonmalignant pediatric case of a gastrosplenic fistula which was treated successfully by surgical intervention.

## Case Report

Our patient is a 16-year-old male with a history of type 1 diabetes mellitus that presented to the emergency department with diabetic ketoacidosis and shock. His blood glucose was 1,037 mg/dL with a pH of 6.95. He was resuscitated, started on an insulin drip, given vasopressors, and admitted to the pediatric intensive care unit (PICU) where he was intubated. During his stay in the PICU, he abruptly had 1.5 L of dark brown to bloody drainage from his nasogastric tube. The dark blown to bloody fluid did not persist and the patient never had a significant drop in his hemoglobin. Had either of these factors developed, an earlier endoscopy would have been warranted. Because of an elevated creatinine (3.4 mg/dL), an abdominal magnetic resonance imaging (MRI) was obtained which showed a small amount of perisplenic fluid and evidence of a splenic infarction. Intravenous antibiotic therapy was initiated with clindamycin. The patient recovered and was discharged from the hospital on day 9.

**Fig. 1:**
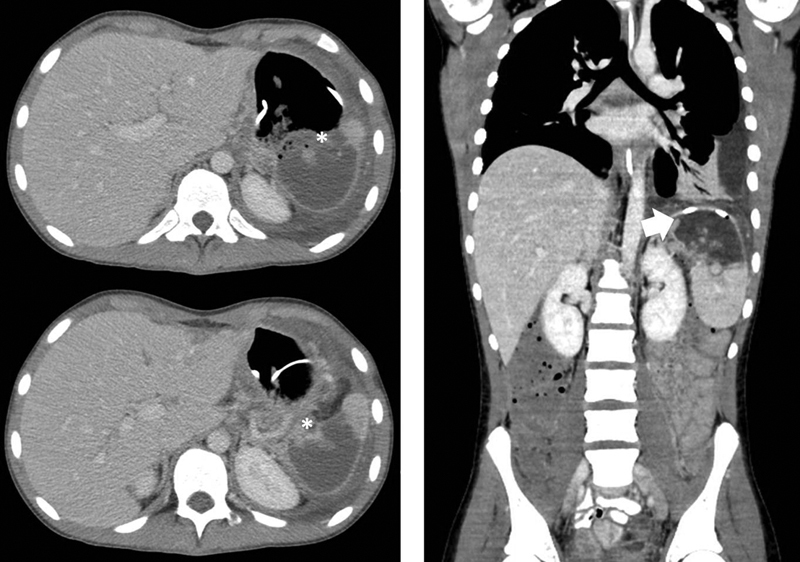
CT scan images demonstrating gastrosplenic fistula. (*) shows fistulous communication between stomach and spleen. Arrowhead represents coiled feeding tube within the capsule of the spleen. CT, computed tomography.

**Fig. 2:**
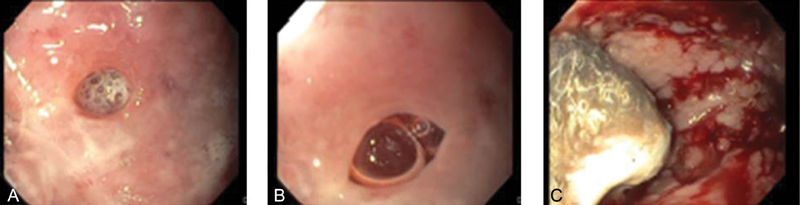
Endoscopic images of gastric inflammation and gastrosplenic fistula. ( **A** and **B** ) Images show pictures of two separate ulcers within there gastric wall. ( **C** ) Image shows generalized immflamation and mucosal erosions of the gastric wall.

The patient presented to the emergency department 2 weeks later with severe epigastric pain, poor appetite, and weight loss of 20 pounds since discharge. He also presented with labored breathing and a chest X-ray showed a left pleural effusion. Initial laboratories were as follows: C reactive protein, 5.5 mg/dL; white blood cell count, 16.10 3 /µL; platelet count, 1,206 × 10 ^3^ /µL; hemoglobin, 9.1 g/dL; and serum lipase 890 U/L. On examination, the patient was ill-appearing and pale with tenderness in the epigastric region. During his hospital stay, the patient's oral intake decreased, and persistent abdominal pain prompted a CT of chest, abdomen, and pelvis with intravenous contrast. The results showed bilateral pleural effusions, left pelvic abscess, pancreatic pseudocyst, and a splenic infarction. Additionally, the computed tomography (CT) was suspicious for a gastrosplenic fistula, as the coil of the patient's transpyloric feeding tube appeared to lie within the upper pole of the spleen ( Fig. 1 ). To further evaluate the possibility of a gastrosplenic fistula, an esophagogastroduodenoscopy was performed, which noted multiple dispersed nonbleeding erosions of the gastric wall with two visualized openings of the posterior gastric wall ( Fig. 2 ). The time between the first and second endoscopy was 3 weeks. There is a growing body of literature to support the use of endoscopic closure of gastric perforations; however, this was not initially considered because of the degree of inflammation and friability of the gastric mucosa.

Due to concern for malignancy, tumor markers were checked and were within normal limits.

A nonoperative approach would have been to support this patient with adequate nutrition and allow for time for the inflammatory process to subside; however, the patient had persistent epigastric pain and feeding intolerance.

The exact etiology of the gastroplenic fistula was not clear and malignancy had not been completely excluded. The history and workup for other causes of a gastrosplenic fistula did not support another etiology. The patient denied a toxic ingestion. Also, none of his imaging on CT scan or MRI showed any evidence of inflammatory bowel disease. He did have an elevated lipase but there were no signs of acute or chronic pancreatitis on his imaging. A positron emission tomography (PET) scan could not be obtained because of the patient's brittle diabetes and insulin requirements. Because of the patient's failure to improve with nonoperative management, surgical exploration was recommended. The operation began with a repeat upper endoscopy to assess the condition of the gastric lumen. However, the scope could not be advanced much beyond the gastroesophageal (GE) junction as the stomach had become very inflamed at this location. Biopsies were not taken at the time of the first endoscopy because of the degree of inflammation and friability of the gastric wall. The changes between the two endoscopies were presumed to be due to progression of the inflammatory process. A left subcostal incision was made and extended across the midline partially to the right subcostal region and extended superiorly to the level of the xiphoid process. Adhesiolysis was performed to take down very dense adhesions between the omentum and left upper quadrant. The anterior gastric wall was intimately attached to the anterior abdominal wall, liver, and spleen. We encountered a very dense inflammatory process involving the upper pole of the spleen. The inflammatory process involving the upper pole of the spleen was able to be divided from the remaining portion of the spleen living the majority of the splenic parenchyma intact. The body of the stomach was completely fibrotic and necrotic. After separating these structures, 4 to 5 cm of proximal stomach beyond the GE junction was found to be viable as was 2 to 3 cm of antrum proximal to the pylorus. Thus, a subtotal gastrectomy was completed by transecting the proximal stomach at the level of viability and transecting the distal stomach at the level of the antrum. A “Roux-en-Y” reconstruction was performed as well as placement of a feeding jejunostomy. Total operative time was 5 hours. The patient initially did well but developed an intra-abdominal abscess in the left upper quadrant seen on a CT that was obtained secondary to fever. The abscess was drained by interventional radiology and found to be from a leak from his gastrojejunal anastomosis. This was managed with external drainage and resolved in 4 weeks. Currently the patient is doing well, has no epigastric pain, and is maintaining his weight on a combination of oral and jejunostomy feeds. Pathological evaluation of the resected stomach revealed marked edema with necrotizing inflammation. There was no evidence of malignancy. A widely patent fistulous tract was seen extending into the congested and hemorrhagic omental tissue.

## Discussion

The etiology of the case is unclear. Possible etiologies include an infarct of the upper pole of the spleen and stomach from a low flow state, complications of pancreatitis, or a toxic ingestion causing necrosis of the stomach. Another cause of this fistula is nasogastric tube trauma; however, this is less likely due to multiple perforations seen on endoscopy. Gastrosplenic fistula can arise as a complication of a primary splenic or gastric malignancy, most commonly diffuse large B cell lymphoma (DLBCL). 2 This may be attributed to this tumor's nature to widely infiltrate the serosa and failure to initiate a desmoplastic reaction. This fistula may occur spontaneously or after recent chemotherapy treatment as a possible consequence of tumor lysis syndrome. 3 Although less frequent benign conditions, such as a benign gastric ulcer, splenic abscess, and Crohn's disease, have also been implicated as a cause. 4 5 6 More recently, a study reported three cases of gastrosplenic fistula as a complication of sleeve gastrectomy. 7 A literature review identified a total of 28 cases reports; the etiology in 75% of cases was determined to be a lymphoma. 8 The most common presenting complaints included abdominal pain (32%), weakness (21%), and upper gastrointestinal bleeding (14%). 8 Abdominal CT is the most useful radiological test for reaching a definitive diagnosis. 9 In a noncontrast CT, an air-fluid level or free air in the spleen may indicate a gastrosplenic fistula, although splenic abscess must be ruled out. Contrasted CT may allow one to visualize the anomalous tract and orally administered contrast may be seen flowing from the stomach to the spleen. Other tests that may be performed include upper GI series as well as upper endoscopy to visualize the opening of the fistula and for tissue sampling.

Surgical resection of the fistula is usually indicated. 9 This is done to prevent leaking of gastric contents that may erode the splenic vessels resulting in life-threatening hematemesis. The operation may include of partial gastrectomy, splenectomy, and/or distal pancreatectomy depending on the underlying etiology. A feeding jejunostomy should also be considered. It is generally done as an open procedure, although a laparoscopic approach has previously been described. 10

## Conclusion

Gastrosplenic fistulas have most commonly been reported in association with splenic and gastric malignancies. Abdominal CT is the most helpful radiological test for diagnosing this because of the ability to show thin cuts and demonstrate very detailed anatomy in this region. Surgical intervention is usually indicated. We have presented a case of gastrosplenic fistula in the pediatric population of unknown etiology that required surgical treatment.

## Notes

**New Insights and the Importance for the Pediatric Surgeon**
Gastrosplenic fistula is a very rare entity most commonly occurring as a distinctive complication of splenic or gastric malignancies, most notably diffuse large B cell lymphoma (DLBCL). We report a nonmalignant pediatric case of a gastrosplenic fistula of unclear etiology and the surgical management.
